# MIB1 mutations reduce Notch signaling activation and contribute to congenital heart disease

**DOI:** 10.1042/CS20180732

**Published:** 2018-12-05

**Authors:** Binbin Li, Liwei Yu, Dong Liu, Xueyan Yang, Yufang Zheng, Yonghao Gui, Hongyan Wang

**Affiliations:** 1 Obstetrics and Gynecology Hospital, State Key Laboratory of Genetic Engineering at School of Life Sciences, Institute of Reproduction and Development, Fudan University, Shanghai 200011, China; 2 NHC Key Lab of Reproduction (Shanghai Institute of Planned Parenthood Research), Collaborative Innovation Center of Genetics and Development, Fudan University, Shanghai 200032, China; 3 Children’s Hospital, Fudan University, Shanghai 201102, China; 4 Co-innovation Center of Neuroregeneration, Key Laboratory of Neuroregeneration of Jiangsu and Ministry of Education, Nantong University, Nantong 226001, China; 5 Institute of Developmental Biology and Molecular Medicine, Fudan University, Shanghai 200433, China; 6 The Institutes of Biomedical Sciences, Fudan University, Shanghai 200032, China

**Keywords:** Congenital heart disease (CHD), Mind bomb 1 (MIB1), Notch signaling pathway, Rare mutations

## Abstract

Congenital heart disease (CHD) is one of the most common birth defects in humans, but its genetic etiology remains largely unknown despite decades of research. The Notch signaling pathway plays critical roles in embryonic cardiogenesis. Mind bomb 1 (Mib1) is a vital protein that activates the Notch signaling pathway through promoting ubiquitination, endocytosis and subsequent activation of Notch ligands. Previous studies show that *Mib1* knockout in mice completely abolishes Notch signaling, leading to cardiac deformity. However, the function of MIB1 and its potential disease-causing mutations are poorly studied in human CHD. In this research, we identified four novel non-synonymous heterozygous rare mutations of *MIB1* from 417 Han Chinese CHD patients. The following biochemical analyses revealed that mutations p.T312K fs*55 and p.W271G significantly deplete MIB1’s function, resulting in a lower level of JAGGED1 (JAG1) ubiquitination and Notch signaling induction. Our results suggest that pathologic variants in *MIB1* may contribute to CHD occurrence, shedding new light on the genetic mechanism of CHD in the context of the Notch signaling pathway.

## Introduction

Congenital heart disease (CHD) is the most common type of birth defect worldwide. It is the leading cause of infant morbidity and disability, seriously decreasing quality of life, while increasing the emotional and financial burden on families and the healthcare system. Embryonic cardiogenesis is delicately regulated by a variety of signaling pathways in time and space. The dysfunction of these signaling pathways could lead to CHD. The evolutionarily conserved Notch signaling pathway plays irreplaceable roles in embryogenesis. It participates in multiple stages of heart development, and its role has been studied in congenital heart disease, cardiomyopathy and adult heart failure [1–5]. Mutations in the Notch signaling pathway have been reported in human congenital heart malformations, such as Alagille syndrome, aortic valve disease and ventricular septal defects [6–10]. The majority of the studies focused on the effects of Notch ligands and their downstream genes. The upstream genes involved in the activation of ligands in Notch signaling pathway are yet to be discussed.

Notch signaling initiation requires Notch ligands (Delta1, 3, 4 and Jagged1, 2 (Jag1, 2) in mammals) intracellular tail ubiquitination and their subsequent endocytosis and activation. This process requires two classes of E3 ubiquitin ligases called Neuralized 1, 2 (Neur1, 2) and Mind bomb 1, 2 (Mib1, 2) [11,12]. Of which, Mib1 is reported to directly interact with and regulate all kinds of Notch ligands, suggesting its broad roles in Notch signaling activation [13]. Repression of Mib1 results in a reduction of Notch signaling activation in mammalian cells [14]. Zebrafish Mib1 mutants exhibit severe defects in somitogenesis, angiogenesis and neurogenesis, accomplished with reduced Notch signaling activity [15–17]. In mice, *Mib1* knockout embryos show complete Notch signaling reduction indicated by repressed Notch active form Notch intracellular domain (NICD) generation, decreased downstream target gene expression, and severely impaired somitogenesis, cardiogenesis, vascular remodeling and accelerated neurogenesis [13]. Unlike the non-redundant roles of Neur and Mib in the fly, only Mib1 is indispensable in mice. Mouse embryos lacking Neur1, Neur2 and Mib2 simultaneously do not show obvious Notch-dependent morphological phenotypes [18–20]. Even though the indispensable role of Mib1 in Notch signaling pathway have been investigated in animal models, data in the context of human health is lacking. So far, only one study reported that MIB1 mutations arrest chamber myocardium development, prevent trabecular maturation and compaction, and result in left ventricular noncompaction cardiomyopathy (LVNC) in humans [21].

Given the evidence for the vital role of MIB1 in early animal cardiogenesis, we hypothesized that genetic variants of *MIB1* would disturb human cardiogenesis and are associated with CHD via disrupted Notch signaling pathway. In order to test our hypothesis, we conducted a case–control study to identify *MIB1* mutations in the sporadic human CHDs. Further functional study indicated a possible mechanism of CHD etiology attributed to *MIB1* genetic variants.

## Materials and methods

### Sample collection and mutation identification

Blood samples from 417 sporadic CHD patients (mean age of 2.9 ± 2.7 years, 55.6% male) were collected from the Cardiovascular Disease Institute of Jinan Military Command (Jinan, Shandong, China). Patients were diagnosed by echocardiography and some were further confirmed surgically. Patients with clinical features of developmental anomalies, positive family history of CHD in a first-degree relative, maternal diabetes mellitus, maternal exposure to known teratogens or any therapeutic drugs during gestation were excluded. For detailed classification of CHD subtypes, please refer to Supplementary Table S1. Blood samples from the 213 ethnically and gender-matched unrelated healthy controls (mean age of 7.1 ± 3.7 years, 49.8% male) were recruited from the same region, and individuals with any congenital anomalies or cardiac disease were excluded. All samples were collected with the approval of local ethics committees and institutional review board of Fudan University. Informed consent documents were signed by the parents or guardians.

Genomic DNA was extracted and targeted exome pooling sequencing of 258 candidate genes was conducted as described previously [22]. Identified variants were filtered using the dbSNP database (https://www.ncbi.nlm.nih.gov/projects/SNP/), the 1000 genomes project (http://www.1000genomes.org), the Exome Aggregation Consortium (ExAC) (http://exac.broadinstitute.org/) and the Genome Aggregation Database (gnomAD) (http://gnomad.broadinstitute.org/). All the case-specific non-synonymous mutations were subsequently confirmed by Sanger DNA sequencing (Supplementary Figure S1).

### Plasmids, cell culture and transfection

pcDNA3.1-HA-MIB1^wild-type^ was constructed by introducing HA epitope sequence and full-length human MIB1 gene coding region (NM_020774.3) into EcoRI/XhoI sites of pcDNA3.1 (ThermoFisher Scientific, #V79020). For pcDNA3.1-HA-MIB1^mutants^, site-directed mutagenesis was carried out with QuikChange Lightning Site-Directed Mutagenesis Kit (Agilent, #210518) according to manufacturer’s instruction. Full-length MIB1 wild-type or mutants without stop codon were subcloned into SgfI/MluI sites of pCMV6-AC-GFP (Origene, #PS100010) to generate pCMV6-AC-GFP-MIB1^wild-type or mutants^. For pCMV6-AC-GFP-MIB1^p.T312K fs*55^, sequence after premature stop codon (including stop codon) was removed for C-terminal tGFP tag fusion. MIB1 mib repeat domain regions (aa 216-462) of wild-type, p.Q237H and p.W271G were inserted into SgfI/MluI sites of pCMV6-AN-Myc (Origene, #PS100012) to obtain pCMV6-AN-Myc-mib reps^wild-type or p.Q237H or p.W271G^. Myc-Flag-tagged full-length human JAG1 (pCMV6-Entry-JAG1) was purchased from Origene (#RC210516). pCMV6-AN-DDK-JICD was generated using JAG1 intracellular domain (aa 1092-1218) subcloned into SgfI/MluI sites of pCMV6-AN-DDK (Origene, #PS100014). TP-1 Luciferase was generated by subcloning synthetic hexamer of Epstein-Barr virus (EBV) terminal protein 1 (*TP1*) gene promoter RBP-Jκ binding sites into KpnI/XhoI sites of pGL3-Basic [23]. pCBFRE Luciferase was purchased from Addgene (plasmid #26897) [24]. Renilla Luciferase reporter pGL4.74[*hRluc*/TK] was purchased from Promega (#E6921). HA-tagged tetrameric ubiquitin expression plasmid (HA-4 Ub) was kindly provided by Dr. Shimin Zhao at Fudan University.

HEK 293T cells were cultured in high-glucose Dulbecco’s Modified Eagle Medium (ThermoFisher Scientific, #11995065) supplemented with 10% FBS at 37°C with 5% CO_2_. Cells were seeded and maintained overnight to reach 80% confluence at the time of transfection. Lipofectamine 3000 Transfection Reagent (ThermoFisher Scientific, #L3000015) was used for transient transfection following manufacturer’s protocol.

### Dual-luciferase reporter assay

Reporter assays were performed with the Dual-Luciferase Reporter Assay System (Promega, #E1910) on GloMax Navigator Microplate Luminometer (Promega, #GM2010). Briefly, each well of 24-well plate of HEK 293T cells was co-transfected with 300 ng of pCDNA3.1-HA-MIB1^wild-type or mutants^, 100 ng of Firefly Luciferase reporters TP-1 Luciferase or pCBFRE Luciferase, and 10 ng of the constitutive Renilla Luciferase reporter pGL4.74[*hRluc*/TK] serving as an internal control. Cells were lysed 24 h post-transfection and assays were carried out according to the manufacturer’s protocol.

### Co-immunoprecipitation and ubiquitination assay

For co-immunoprecipitation of overexpressed proteins, HEK 293T cells seeded in 10 cm dishes were co-transfected with 5 μg of pCMV6-AN-DDK-JICD, and 5 μg of pcDNA3.1-HA-MIB^wild-type or mutants^ or pCMV6-AN-mib reps^wild-type or mutants^. Cells were lysed 24 h post-transfection in lysis buffer (50 mM Tris-HCl, pH 7.5, 150 mM NaCl, 1 mM EDTA, 0.5% Nonidet P-40 and 10% glycerol) supplemented with protease inhibitors (Roche, #11836153001) before use. Immunoprecipitations were conducted with anti-Flag M2 magnetic beads (Sigma, #M8823), and samples were subject to SDS-PAGE and Western Blot with antibodies against Flag (Sigma, #F1804, 1:1000), HA (Sigma, #H3663, 1:1000), Myc (Sigma, #M4439, 1:1000) and GAPDH (Sigma, #G9545, 1:5000). Band density was quantitated by BIO-RAD Image lab software. For detection of ubiquitinated JAG1, 2.5 μg of pCMV6-AC-GFP-MIB1^wild-type or mutants^, 2.5 μg of pCMV6-Entry-JAG1 and 5 μg of HA-4 Ub were co-transfected into HEK 293T cells seeded in 10 cm dishes. Sample preparation and detection were performed as described above with an additional step to pre-treat the cells with 10 μM of MG132 (Sigma, #C2211) for 6 h before harvest.

### Zebrafish embryo injection

pCMV-AC-GFP-MIB1^wild-type or mutants^ linearized by PmeI served as templates. *In vitro* reverse transcription was carried out using mMESSAGE mMACHINE T7 ULTRA Transcription Kit (ThermoFisher Scientific, #AM1345) and the product was cleaned by MEGAclear Transcription Clean-Up Kit (ThermoFisher Scientific, #AM1908). Fertilized eggs were collected from wild-type AB strain zebrafish raised under standard condition. For each group, 400 pg of mRNA was injected into each of ∼200 embryos at 1-2 cell stage. Embryos were collected 72 h post-fertilization and categorized into four groups based on morphology (Normal, wild-type like; Mild, slight bend body axis or pericardial edema; Moderate, severely bend body axis with or without pericardial edema; Severe, body axis does not extend out of yolk ball or severely truncated body axis). Phenotype distribution difference compared with wild-type group was analyzed by Pearson chi-square or Fisher’s exact test.

## Results

### Four missense mutations in *MIB1* gene of CHD patients

**Figure 1:**
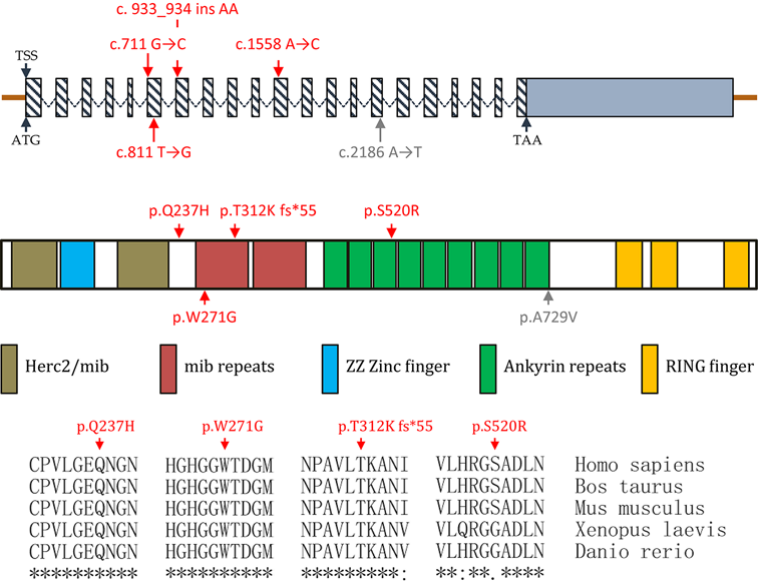
MIB1 Mutations identified in CHD patients and healthy controls Schematic diagram of MIB1 mutations distribution in MIB1 mRNA (NM_020774.3) (upper panel) and protein secondary structure (NP_065825.1) (middle panel), and the partial alignment of MIB1 amino acid among human (NP_065825.1), cattle (NP_001192959.1), house mouse (NP_659109.2), African clawed frog (NP_001085805.1) and zebrafish (NP_775393.2) generated by UniProt online alignment (lower panel). Case-specific mutations are indicated in red and mutation from healthy controls is indicated in gray.

Four case-specific and one control-specific non-synonymous heterozygous mutations (p.Q237H, p.W271G, p.T312K fs*55 and p.S520R in 417 CHD patients, p.A729V in 213 healthy controls) were identified within *MIB1* gene coding region (Figure 1 and Supplementary Table S2), which are absent in dbSNP, the 1000 genomes project, the Exome Aggregation Consortium (ExAC) and the Genome Aggregation Database (gnomAD). Three out of four case-specific mutations map to a defined domain area. Mutation of p.W271G maps to the mib repeat domain involved in substrate interaction. Mutation of p.S520R maps to the ANK repeats domain, which enables MIB1 self-dimerization and substrate interaction [21,25]. The frame-shift mutation of p.T312K fs*55 generates a premature stop codon within one mib repeat domain, resulting in a truncated protein lacking part of mib repeat domains region, ANK repeats domain and the RING finger domains that have E3 ubiquitin ligase activity [25]. Mutation of p.Q237H maps to a location between the second Herc2/mib domain and the first mib repeat domain. Evolutionarily, the sites of p.Q237H and p.W271G are highly conserved across invertebrates to vertebrates, while the site of p.S520R is exclusively conserved among mammals. In addition, mutations of p.Q237H and p.S520R cause amino acid charging alteration, and the mutation of p.W271G inverts the amino acid polarity. Taken together with the prediction by online bioinformatics tools SIFT and POLYPhen-2 (Supplementary Table S2), we predicted that the p.T312K fs*55 is the most deleterious mutation, p.W271G is the second most deleterious mutation, and p.Q237H and p.S520R are minimally damaging or tolerated mutations.

### Mutations affect the MIB1 activity on Notch signaling pathway activation

**Figure 2:**
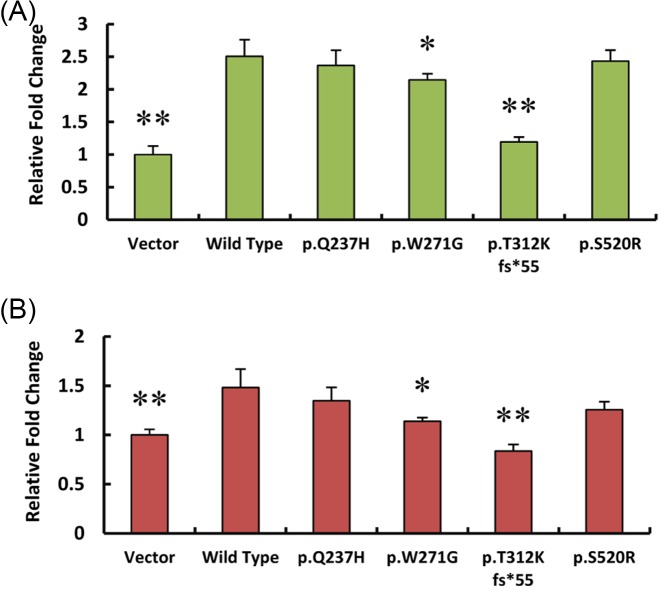
MIB1 mutations affect Notch signaling pathway activation Activation of Notch signaling pathway reporters TP1 Luciferase (**A**) and pCBFRE Luciferase (**B**) by human MIB1 wild-type or mutants 24 h post-transfection in HEK 293T cells. Constitutively expressed Renilla Reniformis Luciferase served as an internal control (*n*≥3. *P<0.05, **P<0.01, compared with wild-type group).

MIB1-induced Notch ligand ubiquitination primes its endocytosis and generation of its activated form from signal sending cells, which subsequently facilitates the cleavage of Notch receptors (Notch 1–4) of signal responding cells [13,26]. NICD is then released and transported into the nuclei to recruit CSL (abbreviation from CBF1/RBP-Jκ, Su(H) and LAG-1) and Mastermind-like based transcription complex and promote downstream target gene expression [11,27]. To test whether MIB1 mutations affect its ability to activate Notch signaling, we performed dual-luciferase reporter assays using two Notch signaling pathway reporters, TP-1 Luciferase and pCBFRE Luciferase. TP-1 Luciferase contains the RBP-Jκ binding sites of EBV *TP1* gene promoter and pCBFRE Luciferase involves EBV CBF1 binding elements. They were separately introduced into HEK 293T cells, along with MIB1 wild-type or mutant expression plasmids, and Renilla Reniformis Luciferase to serve as an internal control. The results showed that wild-type MIB1 dramatically drives Luciferase expression, which indicates Notch signaling activation. However, mutations of p.T312K fs*55 and p.W271G show significantly less luciferase expression, indicating decreased activation of Notch signaling. These data show that both mutations disrupt MIB1 function (Figure 2).

### Mutations alter MIB1 ubiquitination activity on Notch ligand JAG1

**Figure 3:**
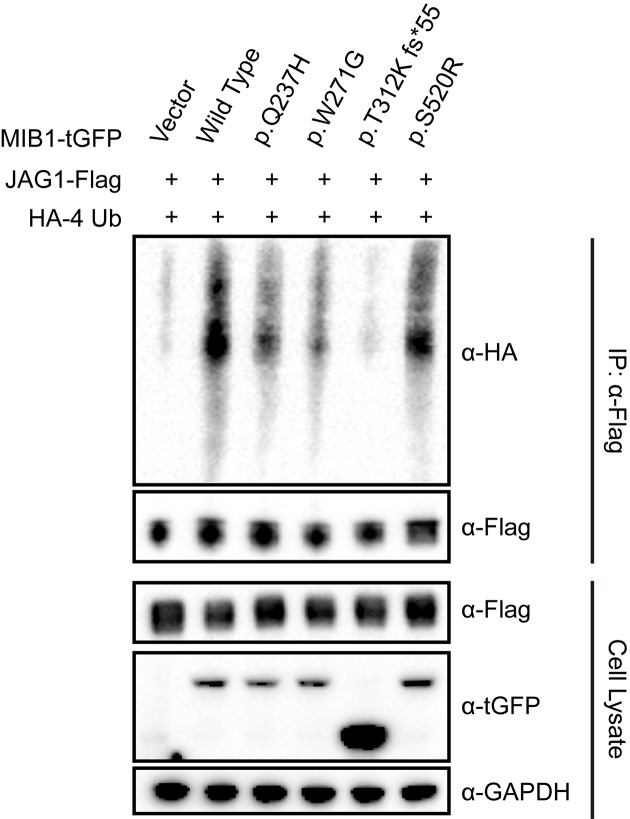
MIB1 mutations affect JAG1 ubiquitination level HEK 293T cells were co-transfected with Flag-tagged full-length JAG1 (JAG1-Flag), tGFP-tagged full-length MIB1 wild-type or mutants (MIB1-tGFP) and HA-tagged tetrameric ubiquitin (HA-4 Ub). Cells were pre-treated with 10 mM MG132 for 6 h before harvest at 24 h post-transfection. Immunoprecipitation was performed with Flag antibody coupled beads, and samples were detected with respective antibodies. Western blot by HA antibody reveals ubiquitination level of JAG1.

Our experiments thus far have demonstrated that some mutations in MIB1 affect Notch signaling pathway activation. Now that MIB1 activates the pathway through modifying substrates, we hypothesized that these mutations would alter the ubiquitination level of Notch ligands. To test this, HEK 293T cells were co-transfected with Flag-tagged full-length JAG1, tGFP-tagged full-length MIB1 wild-type or mutants and HA-tagged tetrameric ubiquitin. As shown in Figure 3, JAG1 ubiquitination is strongly up-regulated in cells co-transfected with wild-type MIB1 compared with empty vector. However, p.T312K fs*55 mutation nearly abolishes the ubiquitination of JAG1. In addition, p.W271G mutation causes a significant decline of ubiquitinated JAG1. Slight change and no obvious difference were found with p.Q237H and p.S520R mutations, respectively. Thus, our results indicate that p.T312K fs*55 and p.W271G mutations strongly impair MIB1 function on substrate ubiquitination.

### Mutations influence the interaction of MIB1 with JAG1

**Figure 4:**
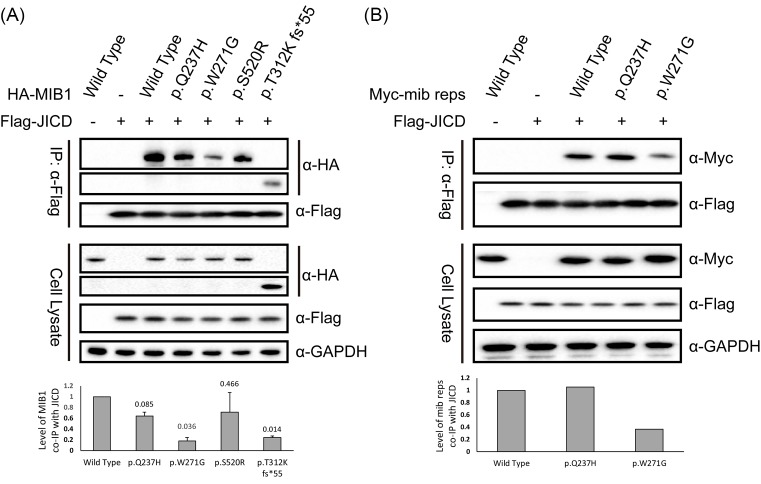
MIB1 mutations affect its interaction with JAG1 HEK 293T cells were co-transfected with Flag-tagged JAG1 intracellular domain (Flag-JICD) and HA-tagged full-length MIB1 wild-type or mutants (HA-MIB1) (**A**, statistic assay of band density was calculated from two independent experiments) or Myc-tagged mib repeats domain region of MIB1 wild-type or variants (Myc-mib reps) (**B**). Twenty-four hours post-transfection, cell lysate was immunoprecipitated with Flag antibody immobilized beads, and samples were detected with respective antibodies.

It is not surprising that the frame-shift mutation of p.T312K fs*55 affects MIB1 function since it results in the loss of the enzymatic RING finger domains and most parts of the interacting domains. As for the other mutations, one possibility for the function loss is that they influence the interaction of MIB1 with substrates since they either are in or flank the interacting domains (Figure 1). To test our hypothesis, Co-IP of full-length or truncated MIB1 wild-type or mutants with JAG1 intracellular domain (JICD) was conducted using HEK 293T cells. As shown in Figure 4A, interaction between MIB1 and JAG1 is severely interrupted by p.T312K and p.W271G mutations. Similar phenomenon for p.W271G was observed when using truncated MIB1 (mib repeat domains region) (Figure 4B), thus supporting our hypothesis that this mutation impairs MIB1 function through disturbing the interaction of MIB1 with its substrates.

### Assessment of human MIB1 wild-type or mutant overexpression on zebrafish embryogenesis

**Figure 5:**
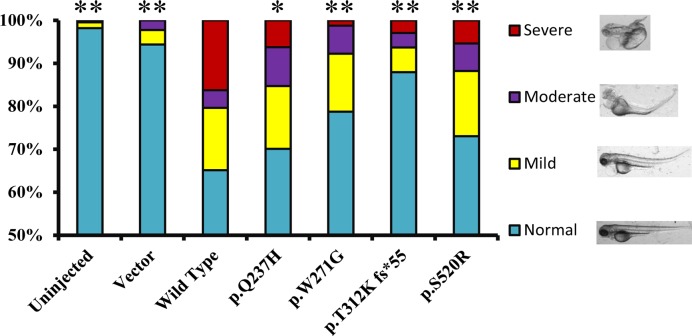
Effect of overexpressed MIB1 wild-type or mutant on zebrafish development In vitro synthesized mRNA was microinjected into 1-2 cell stage Fertilized zebrafish eggs and the teratogenic effect was analyzed 72 h post-fertilization based on phenotypes. Bars with colors show the distribution of four categories of normal and different degree of dysmorphic phenotypes (*P<0.05, **P<0.01, compared with wild-type group)

The Notch signaling pathway is evolutionarily conserved, which plays fundamental roles in organogenesis, particularly of the embryonic heart. Given that, it is not surprising that both gain and loss of function in the Notch signaling pathway influence embryo development and lead to congenital malformations. Hence, we performed mRNA microinjection into one cell or two cells stage fertilized zebrafish eggs to evaluate the teratogenic effect of human MIB1 overexpression. Seventy-two hours post-fertilization, embryos were clustered into four groups based on the degree of malformation. Compared with the un-injected and empty vector groups, wild-type MIB1 group resulted in the most severe embryonic malformations, while the other mutations resulted in less severe teratogenic effects than wild-type MIB1 statistically (Figure 5 and Supplementary Table S3). The first and second least aberrant groups of embryos were generated by p.T312K fs*55 and p.W271G, respectively, indicating that the function of MIB1 protein is most severely damaged by these two mutations.

## Discussion

In the present study, we focused on the obligatory Notch ligand E3 ubiquitin ligase gene *MIB1* and identified four heterozygous missense rare mutations specifically existing in CHD patients. Both *in vitro* and *in vivo* functional analyses demonstrated that the frame-shift mutation of p.T312K fs*55 and the substitutive mutation p.W271G significantly impair MIB1 function in terms of substrate affinity and ubiquitination level, and subsequent Notch signaling pathway activation. The other two substitutive mutations of p.Q237H and p.S520R also demonstrate mild dysfunction in some of the assays. To our knowledge, this is the first study to report functional MIB1 mutations in human CHD and their possible mechanism.

In fact, MIB1 takes part in several cellular processes other than the Notch signaling pathway, such as the Wnt/β-Catenin signaling pathway, which is also a vital signaling pathway in cardiogenesis [28–32]. It was reported that MIB1 positively regulates this pathway through ubiquitination of receptor-like tyrosine kinase (RYK) [29]. The interacting region of MIB1 with RYK differs from that with JAG1. According to the study, the N terminus of MIB1 (aa 1-124), rather than the regions of mib repeats and ANK repeats, interacts with RYK intracellular domain. Since none of the identified mutations map to that essential area, we did not perform any experiment to test whether the mutations would interfere with MIB1’s ability to activate Wnt/β-Catenin signaling pathway. Nevertheless, it is worth noting that those mutations may also impair MIB1 function in the context besides Notch and Wnt signaling pathways.

Further investigation into the clinical data from the patients with these four mutations revealed that three of the patients also carry case-specific missense mutations of LTBP4, which is a member in TGF-β signaling pathway. A fourth patient with the p.Q237H mutation did not carry this additional LTBP4 mutation. Taken together with our functional data that the p.Q237H mutation causes relatively mild damage to MIB1 function, it might explain why this patient has a relatively mild form of CHD, which is a patent ductus arteriosus (Supplementary Table S2). There is limited method to test this idea, but the effect of combined mutations should be considered in a polygenic disease like CHD.

This case–control study revealed the association between harmful mutations of MIB1 with CHD risk. Yet, due to limitations in sample collection and DNA availability from children’s parents, we were unable to represent our data in pedigrees. In fact, the conventional pedigree analysis is often inaccessible. Moreover, variant co-segregated with the disease in pedigrees is not always identifiable, especially in the multi-gene disease like CHD. Thus, identifying deleterious rare mutations of the core cardiogenic genes in sporadic patients could potentially provide a breakthrough in understanding the genetic etiology of CHD. A well-established patient-specific genetic variant pool will not only deepen our knowledge of this disease, but contribute to a risk assessment model that could guide early prenatal diagnosis and intervention as well.

To summarize, we identified four non-synonymous heterozygous rare mutations of *MIB1* in Chinese Han population. Functional studies both *in vitro* and *in vivo* showed that some of the mutations distinctly impair the activity of MIB1 in the Notch signaling pathway. Our research provides new information regarding the role of MIB1 in cardiogenesis and the molecular mechanism of CHD pathogenesis.

## Clinical perspectives

- Our study provided the first evidence that functional MIB1 mutations are associated with human CHD through the Notch signaling pathway. The present study deepened our understanding of this pathway, especially the function of its upstream that is not fully investigated in humans previously.
- The novel missense mutations p.T312K fs*55 and p.W271G are loss-of-function mutations, which impair Notch ligand binding and subsequent activation. Attenuated signaling induction was observed both *in vitro* and *in vivo*, indicating that MIB1 malfunction could disrupt heart development.
- Our results enriched the knowledge of CHD etiology and the genetic variant pool, which potentially provide theoretical basis for early prenatal diagnosis and intervention of human CHD.

## Supporting information

**Table S1.** Classification of CHD subtypes in our study

**Table S2.** Summary of mutations and clinical information of the carriers

**Table S3.** Phenotypic statistics of zebrafish embryo injection

## Abbreviations

CHD: congenital heart disease
MIB1: Mind bomb 1
JAG1: JAGGED1
Neur1, 2: Neuralized 1, 2
LVNC: left ventricular noncompaction cardiomyopathy
EBV TP1: Epstein-Barr virus terminal protein 1
ExAC: Exome Aggregation Consortium
gnomAD: Genome Aggregation Database
NICD: Notch intracellular domain
CSL: CBF1/RBP-Jκ, Su(H) and LAG-1
MAML: Mastermind-like
JICD: JAG1 intracellular Domain
RYK: receptor-like tyrosine kinase
