# Supplementary material for: MIB1 mutations reduce Notch signaling activation and contribute to congenital heart disease
Source: Clin Sci (Lond). 2018 Dec 5;132(23):2483–91. doi: 10.1042/CS20180732 (PMC6365626; doi:10.1042/CS20180732)
Supplement: Supplementary file 1 [file cs-132-cs20180732_supp1.pdf]

Table S1. Classification of CHD subtypes in our study

Diagnosis	Number
Conotruncal	139 (33.3%)
Septal[a]	138 (33.1%)
RVOTO	46 (11.0%)
AVSD	22 (5.3%)
LVOTO	17 (4.1%)
APVR	12 (2.9%)
Complex[b]	10 (2.4%)
Heterotaxy	3 (0.7%)
Others[c]	30 (7.2%)
**Total**	**417 (100%)**

AVSD：Atrioventricular septal defect; APVR: Anomalous pulmonary venous return; LVOTO: Left Ventricular outflow tract obstruction; RVOTO: Right ventricular outflow tract obstruction; PDA: Patent ductus arteriosus.

[a] Group “Septal” includes VSD, ASD (Except ASD I) etc.

[b] Group “Complex” includes single ventricle, L-TGA and multiple complex heart anomalies.

[c] 387 out of 417 cases were classified into first eight groups according to the method introduced by Lorenzo et al. [1]. Whereas 30 cases can’t be classified into the study mentioned above and are sorted into “Other” groups, which includes 17 cases of isolated PDA, 8 cases of aproctia with CHD, 2 cases of mitral insufficiency, 2 cases of aortopulmonary window and 1 mitral stenosis.

1. Botto, L.D., A.E. Lin, T. Riehle-Colarusso, S. Malik, A. Correa, and S. National Birth Defects Prevention, *Seeking causes: Classifying and evaluating congenital heart defects in etiologic studies.* Birth Defects Res A Clin Mol Teratol, 2007. **79**(10): p. 714-27.

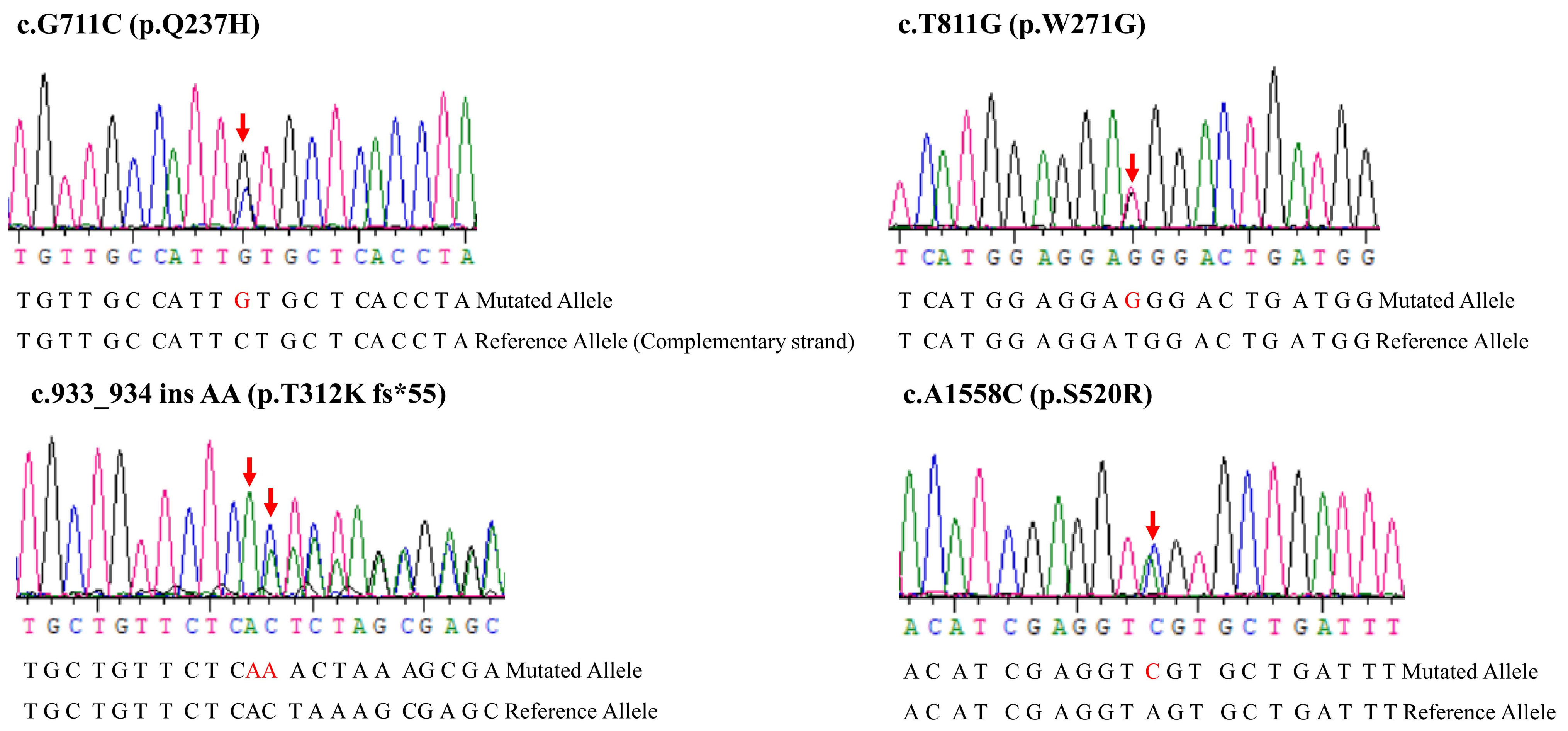
c.G711C (p.Q237H)
T G T T G C CA T T G T G C T CA C C T A Mutated Allele
T G T T G C CA T T C T G C T CA C C T A Reference Allele (Complementary strand)
c.T811G (p.W271G)
T CA T G G A G G A G G G A C T G A T G G Mutated Allele
T CA T G G A G G A T G G A C T G A T G G Reference Allele
c.933_934 ins AA (p.T312K fs*55)
T G C T G T T C T CAA A C T A A AG C G A Mutated Allele
T G C T G T T C T CAC T A A A G CG A G C Reference Allele
c.A1558C (p.S520R)
A C A T C G A G G T C G T G C T G A T T T Mutated Allele
A C A T C G A G G T A G T G C T G A T T T Reference Allele

Table S2 Summary of mutations and clinical information of the carriers

Nucleotide [a]	Amino Acid [b]	SIFT	POLYPhen-2	Age	Gender	Diagnosis
c.G711C	p.Q237H	Tolerated	Benign	1.3	Male	PDA
c.T811G	p.W271G	Deleterious	Possibly damaging	7.75	Male	VSD, ASD, PDA
c.933_934 ins AA	p.T312K fs*55	N/A	N/A	3	Female	ASD, PS
c.A1558C	p.S520R	Deleterious	Benign	5.5	Female	ASD, PS
c.C2186T	p.A729V	Tolerated	Benign	11	Female	Healthy control

[a] NM_020774.3; [b] NP_065825.1.

ASD: Atrial septal defect; VSD: Ventricular septal defect; PDA: Patent ductus arteriosus; PS: Pulmonary Stenosis.

Table S3 Phenotypic statistics of zebrafish embryo injection

	**Normal**	**Mild**	**Moderate**	**Severe**	**n**	***p*(vs WT)**	
**Uninjected**	221	3	1	0	225		
**Vector**	168	6	4	0	178		
**Wild Type**	112	25	7	28	172		
**p.Q237H**	101	21	13	9	144	*	Pearson
**p.W271G**	193	33	16	3	245	**	Pearson
**p.T312K fs*55**	182	12	7	6	207	**	Fisher
**p.S520R**	217	45	19	16	297	**	Pearson

*p<0.05; **p<0.01
